# Signs, symptoms and biochemistry in recurrent Cushing disease: a prospective pilot study

**DOI:** 10.1007/s12020-021-02719-9

**Published:** 2021-04-19

**Authors:** Leah T. Braun, Stephanie Zopp, Frederick Vogel, Jürgen Honegger, German Rubinstein, Katharina Schilbach, Heike Künzel, Felix Beuschlein, Martin Reincke

**Affiliations:** 1 Medizinische Klinik und Poliklinik IV, LMU Klinikum, München, Germany; 2 Department for Neurosurgery, University Hospital Tübingen, Tübingen, Germany; 3 Klinik für Endokrinologie, Diabetologie und Klinische Ernährung, Universitätsspital Zürich, Zurich, Switzerland

Cushing disease (CD) is a rare endocrine disorder caused by ACTH secretion leading to cortisol excess [1]. Reported recurrence rates are quite high throughout most of the studies [2, 3]. Usually, close surveillance after first successful therapy is recommended [4–6], including both clinical and biochemical assessment. The Endocrine Society Clinical Practice Guideline on the treatment of CS recommends to screen patients for recurrence after recovery of the HPA axis and after that, annually or if the patient has clinical symptoms [5]. Low-dose dexamethasone suppression test, late-night salivary cortisol and cortisol in a 24-h collection are part of the standard screening approach. Of these tests, midnight salivary cortisol is the first parameter to become abnormal in patients with recurrence [7–9].

Currently, there is a level of uncertainty in the diagnosis of recurrence as on one side, a cyclic biochemical pattern is not uncommon in recurrent CS [10], which makes it challenging to confirm suspected recurrence by biochemical screening. On the other side, physiological forms of hypercortisolism may also occur in patients who are actually in remission [11]. Based on this background, we analyzed prospectively the clinical and biochemical course of patients with recurrent CD to answer the question, in which sequence clinical and biochemical parameters recur. Using a historic retrospective cohort for comparison, we wanted to answer whether annual follow-up identifies recurrence earlier.

## Materials and methods

### Patients, procedures and variables

This study is part of the prospective German Cushing Registry (founded in 2012). So far, 67 prospectively studied patients with newly diagnosed overt CD formed the study cohort. Furthermore, 100 patients with treated CD were retrospectively included in the registry at variable time points of their disease. These patients did not receive a structured annual follow-up assessment. The German Cushing Registry was approved by the LMU ethics committee, and all patients gave written informed consent.

Successful surgery was defined as a phase of adrenal insufficiency post-surgery, requiring glucocorticoid replacement therapy, and a regression of clinical symptoms. Patients were examined 1, 3, 6 and 12 months after surgery and annually thereafter (checklist-based clinical examination, recording of new or worsening comorbidities combined with biochemical testing by 1-mg low-dose dexamethasone suppression test, urinary free cortisol and late-night salivary cortisol). Depending on the clinical and biochemical results, patients are classified as being in remission or as having suspected recurrence.

### Biochemical evaluation

All patients underwent blood samples between 8:00 and 11:00 am in the fasting state. Patients collected late-night saliva and urine over 24 h at home (one morning urine was included in the total volume). LDDST was performed after sampling of urine and saliva. Urinary cortisol was measured at the central laboratory of our hospital using an automated electro-chemiluminescence immunoassay (LIAISON DiaSorin, Saluggia, Italy, reference value <85 µg/24 h, grey zone 85–115 µg/24 h). Patients receiving glucocorticoid replacement therapy at time of evaluation paused hydrocortisone for 16 h before starting biochemical sampling.

All other laboratory analyses were performed at the Endocrine Laboratory of the Medizinische Klinik und Poliklinik IV, LMU Munich. Salivary cortisol, serum cortisol and plasma ACTH were measured using automated CLIAs, (salivary cortisol: IDS-iSYS, Immunodiagnostic Systems, Boldon, UK; serum cortisol and plasma ACTH: LIAISON DiaSorin, Saluggia, Italy), as reported previously [12]. Normal late-night salivary cortisol is defined as <1.5 ng/mL (grey zone >1.5 <2.3 ng/mL).

### Statistics

For statistical analysis, SPSS 26 was used. Non-parametric tests were used to test differences between groups. *P* values of < 0.05 were considered to indicate statistical significance.

## Results

Of the 67 patients, 14 were excluded due to incomplete data/loss to follow-up. Of the remaining 53 patients, 9 had persistent disease after first surgery. Nine of the 44 patients who were in remission after first surgery developed biochemical or clinical abnormalities during a mean follow-up time of 46 ± 20 month. All nine patients (5 women, 4 men) suffered from adrenal insufficiency within the first 4 weeks post surgery (median baseline cortisol: 4.6 (1.0–9.0) µg/dL).

As a general pattern we did not observe clinical signs of CS without biochemical abnormalities. Compared to biochemical and clinical results at first diagnosis, all variables were significantly lower at time of recurrence (*p* = 0.01). The main symptom in the recurrence group was weight gain in 56% of patients. Comorbidities were more common at first diagnosis than in recurrent disease (*p* = 0.03). Table 1 shows clinical and biochemical variables.

**Table 1:** Course of clinical signs, comorbidities and biochemical variables at time of first diagnosis and at time of recurrence (*N* = 9) of the prospective Cushing cohort

	First diagnosis	Recurrence	*p* values
Clinical symptoms (frequency)			
Weight gain	100%	56%	
Buffalo hump	89%	11%	
Moon face	78%	None	
Bruises	68%	None	
Plethora	67%	None	
Muscle weakness	67%	11%	
Loss of libido	67%	None	
Odema	56%	11%	
Thin skin	44%	22%	
Hair loss	44%	None	
Hirsutism	22%	None	
Acne	11%	None	
Mean number of symptoms	7.0 ± 3.4	1.1 ± 1.2	***p*** = **0.01**
Biochemical screening (median and quartiles)			
9 am serum cortisol (µg/dL) reference interval: 4–24 µg/dL	38 (20–45)	10 (8–11)	***p*** = **0.01**
ACTH (pg/mL) reference interval: 10–50 pg/ml	96 (50–109)	34 (16–47)	***p*** = **0.02**
LDDST (µg/dL) reference interval: <2	18 (15–27)	4 (3–6)	***p*** = **0.01**
Late-night salivary cortisol (ng/mL) reference interval: <1.5 nmol/L	12 (5–22)	4 (2–5)	***p*** = **0.01**
UFC (µg/24 h) reference interval: <85 µg/die	748 (394–1220)	111 (70–253)	***p*** = **0.01**
Clinical examination (median and quartiles)			
BMI	27 (23–32)	24 (23–28)	*p* = 0.2
Hip (circumference in cm)	97 (94–101)	100 (98–102)	*p* = 0.5
Waist (circumference in cm)	110 (86–115)	97 (76–100)	***p*** = **0.04**
Upper arm (circumference in cm)	27 (26–30)	27 (26–30)	*p* = 0.6
Waist-hip-ratio	1.0 (0.97–1.15)	0.96 (0.80–0.97)	***p*** = **0.03**
Waist-height-ratio	0.59 (0.51–0.71)	0.51 (0.44–0.61)	***p*** = **0.05**
Waist-arm-ratio	3.6 (3.5–4.1)	3.2 (2.9–3.3)	***p*** = **0.03**
Blood pressure (systolic in mmHg)	149 (140–168)	129 (123–133)	*p* = 0.07
Blood pressure (diastolic in mmHg)	99 (87–111)	84 (78–89)	*p* = 0.06
Comorbidities (frequencies)			
Hypertension	100%	33%	
Diabetes mellitus	22%	11%	
Dyslipidemia	67%	44%	
Osteoporosis	44%	22%	
Depression	22%	22%	
Number of comorbidities	2.7 ± 0.9	1.4 ± 0.9	***p*** = **0.03**

Bold numbers indicate significance

*UFC* urinary free cortisol, *LDDST* 1-mg low-dose dexamethasone suppression test, *TSS* transsphenoidal surgery, *LNSC* late-night salivary cortisol

Mean interval time between first diagnosis and diagnosis of recurrence was 49 months (±22). In six of the nine patients, biochemical parameters were abnormal before patients developed signs and symptoms of CS (individual pattern see Supplementary Fig. 1). In the other three patients, clinical symptoms and biochemical abnormalities occurred at the same time. In three of the nine patients, all three tests turned abnormal at the same time, in the other six patients, one or two tests turned abnormal first. Treatment at first diagnosis was surgery in all patients. Patients with recurrence received medical treatment (*n* = 3), second transsphenoidal surgery (*n* = 1), radiotherapy (*n* = 1) and no treatment (*n* = 4, ‘wait-and-watch’ strategy).

### Historic control cohort

Sixteen patients of the historic retrospective cohort had recurrence of CD. Recurrence was diagnosed significantly later than in the prospective cohort (mean 8.6 ± 4.8 vs. 4.2 ± 1.6 years of follow-up, *p* < 0.048). Urinary free cortisol was higher and clinical signs and symptoms were more common and more pronounced (Supplementary Table 1). All of these patients received a treatment (no ‘watch-and-wait’ strategy).

## Discussion

Regular follow-ups after first successful surgery are recommended [5, 13, 14] but a unifying consensus on specific clinical and biochemical testing and follow-up intervals has not been established so far. Also, recurrence criteria are not universally agreed on [15].

In our study, we pursued an exploratory approach: as we observed our patients prospectively, we collected in parallel complete clinical and biochemical data of patients with CD. A major finding of our study is that the prospective assessment identified patients with recurrent CD much earlier (4 vs. 8 years). Moreover, biochemical activity, the Cushingoid phenotype and comorbidities were much more prominent in the latter than in the former.

It is the essence of this approach that patients with clinical signs of CD always have abnormal biochemical tests but not the other way round. Therefore, a clinical approach with focus on signs and symptoms will lead to a delayed diagnosis of recurrence, compared to annual biochemical screening. However, the early diagnosis resulted into a watch-and-wait strategy in nearly half of our patients. Whether this scenario is of advantage for patients or has negative impact in terms of quality-of-life outcomes cannot be answered in our study. Other studies suggest to start a specific treatment early, in analogy to patients with subclinical adrenal CS, in whom hypercortisolism is associated with increased cardiovascular morbidity and mortality [16]. A study by Carroll et al. emphasized that patients with recurrence can benefit from early secondary treatments [17]. A theoretical advantage of early diagnosis of recurrence might lay in a higher flexibility regarding treatment options, using pasireotide in patients with mild hypercortisolism [18, 19] or radiotherapy without the need for a bridging hypercortisolism-directed pharmacotherapy.

### Limitations, strengths and outlook

This study has limitations including the monocentric design and the quite small sample size of patients with recurrence. This contrasts with its strength, the comprehensively, prospective observation of the study cohort. To our knowledge, there is no other study reporting the course of recurrence in a similar way. Future studies should especially focus on the benefits of an early diagnosis or recurrence and the potential influence on the long-term outcome of patients with CD.

## Supplementary information

Table 1_Supplement

Supplement Figure 1

## Data Availability

All data used for this study are included in the manuscript.
