# Supplementary material for: Signs, symptoms and biochemistry in recurrent Cushing disease: a prospective pilot study
Source: Endocrine. 2021 Apr 19;73(3):762–6. doi: 10.1007/s12020-021-02719-9 (PMC8325659; doi:10.1007/s12020-021-02719-9)
Supplement: Supplementary file 1 — Table 1_Supplement [file 12020_2021_2719_MOESM1_ESM.docx]

**Supplement**

**Table 1: Validation cohort –patients without structured follow-up and recurrence. Shown are clinical and biochemical parameters and co-morbidities at time of recurrence (N = 16), median and quartiles or frequencies**

| **Biochemical** | | **Comorbidities** | |
| --- | --- | --- | --- |
| **Serum cortisol** (µg/dL)  *reference interval: 4-24µg/dL* | 12 (8 – 17) | **Hypertension** | 44% |
| **ACTH** (pg/mL)  *reference interval: 10-50pg/ml* | 41 (27 – 59) | **Diabetes mellitus** | 19% |
| **LDDST** (µg/dL)  *reference interval: < 2* | 7 (3 – 12) | **Dyslipidemia** | 19% |
| **Late-night salivary cortisol** (ng/mL)  *reference interval: <1.5nmol/L* | 4 (3 – 12) | **Osteoporosis (T-Score <2.5)** | 38% |
| **UFC** (µg/24h)  *reference interval: < 85 µg/die* | 236 (175 – 738) | **Major depression** | 25% |
| **Clinical Signs** | | **Clinical examination** | |
| **Weight gain** | 38% | **BMI** | 27 (22 – 32) |
| **Moon face** | 19% | **Hip (size in cm)** | 103 (95 – 109) |
| **Plethora** | 44% | **Waist (size in cm)** | 96 (79 – 110) |
| **Acne** | 13% | **Upper arm (size in cm)** | 30 (26 – 32) |
| **Buffalo hump** | 25% | **Waist-hip-ratio** | 0.95 (0.77 – 0.97) |
| **Thin skin** | 25% | **Waist-height-ratio** | 0.55 (0.46 – 0.66) |
| **Bruises** | 25% | **Waist-arm-ratio** | 3.2 (2.7 – 3.6) |
| **Hair loss** | 25% | **Blood pressure (systolic in mmHg)** | 138 (119 – 150) |
| **Hirsutism** | 6% | **Blood pressure (diastolic in mmHg)** | 87 (76 – 93) |
| **Muscle weakness** | 19% |  |  |
| **Loss of libido** | 31% |  |  |
