# Supplementary figures and images for: Signs, symptoms and biochemistry in recurrent Cushing disease: a prospective pilot study
Source: Endocrine. 2021 Apr 19;73(3):762–6. doi: 10.1007/s12020-021-02719-9 (PMC8325659; doi:10.1007/s12020-021-02719-9)

**Supplement Figure 1**


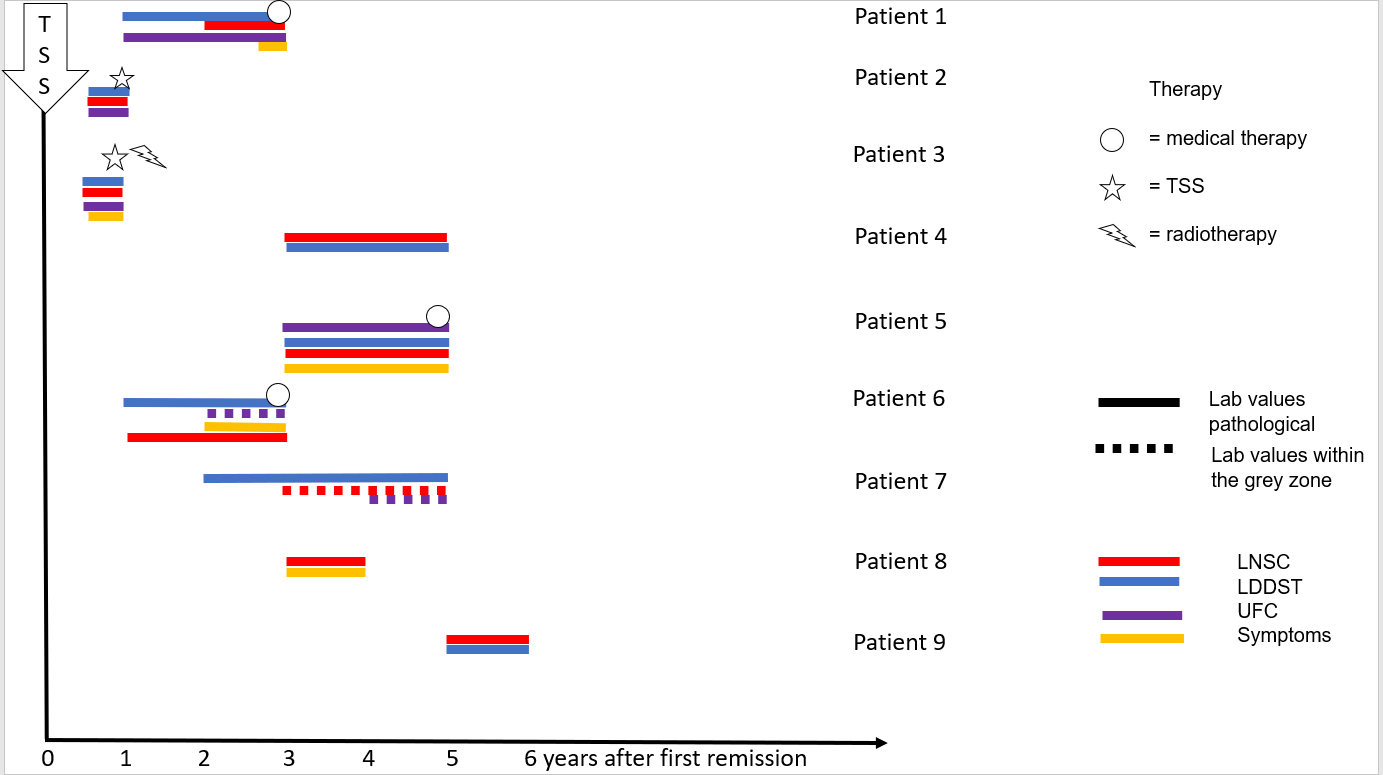

Supplement: Supplementary file 2 — Supplement Figure 1 [file 12020_2021_2719_MOESM2_ESM.docx]
